# Fibroblast Growth Factor-Derived Peptides: Sources, Functions, and Applications

**DOI:** 10.3390/bioengineering12101019

**Published:** 2025-09-25

**Authors:** Cheng-Kun Cao, Zhong-Yuan Shi, Chuan-Bang Chen, Xiao-Kun Li, Zhi-Jian Su

**Affiliations:** 1 School of Pharmaceutical Sciences, Wenzhou Medical University, Wenzhou 325000, China; 2 Department of Rehabilitation, The First Affiliated Hospital of Wenzhou Medical University, Wenzhou 325000, China; 3 National Engineering Research Center for Cell Growth Factor Drugs and Protein Biologics, Wenzhou 325000, China; 4 National Key Laboratory of Macromolecular Drug Development and Manufacturing, Wenzhou 325000, China

**Keywords:** fibroblast growth factor-derived peptides, function, clinical application

## Abstract

Fibroblast growth factors (FGFs) play a crucial role in various biological processes, including tissue development, metabolic regulation, and injury repair. Previous studies have shown that certain peptides can exhibit similar biological functions to FGFs, whether they are fragments extracted from natural FGF molecules or derived peptides designed based on the structural characteristics of FGFs and their receptor molecules. These FGF-derived peptides have shown significant application potential in fields including tissue repair and regeneration, cancer therapy, metabolic regulation, neural recovery, and biological delivery. This article reviews the sources, bioactive functions, molecular mechanisms, and application prospects of FGF-derived peptides, aiming to provide new research ideas for subsequent structural optimization, drug delivery system development, and clinical translation of these peptides.

## 1. Introduction

**Figure 1:**
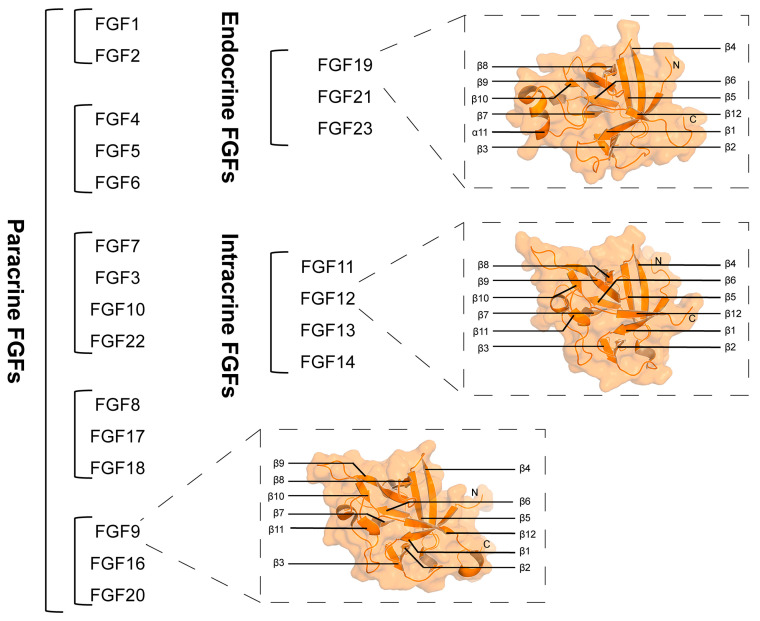
A brief overview of FGFs. There are 22 identified FGFs, which are classified into seven subfamilies. Structure diagrams of FGF9 (PDB ID: 5W59), FGF12 (PDB ID: 1Q1U) and FGF19 (PDB ID: 2P23).

As a highly conserved protein family during biological evolution, fibroblast growth factors (FGFs) play a crucial role in embryonic development, tissue regeneration and repair, as well as the maintenance of metabolic homeostasis [1,2,3,4]. Since FGF1 was first isolated from the bovine pituitary in 1973 [5], the number of FGF family members has now expanded to 23, and 22 of them are encoded by the human genome (FGF15 in mice) [6]. Based on their sequence homology, FGFs can be classified into seven subfamilies, including the FGF1 subfamilies, the FGF4 subfamilies, the FGF7 subfamilies, the FGF8 subfamilies, the FGF9 subfamilies, the FGF11 subfamilies, and the FGF19 subfamilies [7]. According to their different modes of action, these seven subfamilies can be further divided into paracrine type (FGF1/4/7/8/9 subfamilies), endocrine type (FGF19 subfamily), and intracellular type (FGF11 subfamily) [8] (Figure 1). The molecular weights of FGF family members range from 17 to 34 kDa, and their sequence homology ranges from 13% to 71% [9]. The core regions of paracrine and intracellular FGFs are both composed of 12 parallel β strands, which fold to form a typical β-trifolium structure. Among them, the 10th and 11th β strands play a crucial role in promoting the binding of FGFs to heparan sulfate (HS). In contrast, because endocrine FGFs lack the 11th β chain, they are unable to form a complete β-trifolium structure; thus, their affinity for HS is significantly reduced [10,11,12,13] (Figure 1). Compared with the highly conservative core region, the N-terminal and C-terminal sequences of FGFs are highly diverse. This characteristic endows the FGF family proteins with rich functional properties [14].

Recent studies have shown that, obtained by extracting the natural sequences of FGFs or rationally designed based on the interaction interface between FGFs and their receptors (fibroblast growth factor receptors, FGFRs), some short peptides consisting of 4 to 28 amino acid residues can mimic or antagonize the biological functions of full-length FGFs. These short peptides are collectively referred to as FGF-derived peptides. Compared to full-length FGFs, FGF-derived peptides possess several advantages, including structural simplicity, low cost, ease of storage, low immunogenicity, high tissue permeability, and facile conjugation with biomaterials [15,16,17]. Currently, both naturally occurring and artificially designed FGF-derived peptides have made significant progress in the fields of tissue repair and regeneration, targeted disease therapy, and the development of novel biological delivery technologies (Table 1). They have gradually overcome, to some extent, the limitations of traditional FGF protein drugs in clinical applications [18]. Therefore, FGF-derived peptides hold great promise as an important direction for the development of next-generation therapeutic molecules.

This article provides a systematic review of the current research progress of FGF-derived peptides. The content covers their molecular sources, functional mechanisms, and applications in the treatment of various diseases. Although there are numerous research reports and discussions on related directions, providing highly valuable content, our review also offers a unique perspective, proposing a potential dual-mode mechanism of FGF’s action in the body. Specifically, we discuss that FGF may not only exert its effect by binding to FGFR in its full-length form to initiate downstream signal cascades but also may be hydrolyzed into biologically active short peptides. These peptides may have stronger tissue permeability and play a role in maintaining physiological homeostasis through finely regulatory effects. To our knowledge, this dual-action hypothesis has not been explicitly emphasized in previous reviews, which is precisely the uniqueness of this study. Additionally, the article also discusses the key challenges and future development directions currently faced by FGF-derived peptides. The aim is to provide new ideas for their further research and clinical translation.

## 2. Molecular Basis of the Functional Activity of FGF-Derived Peptides

**Figure 2:**
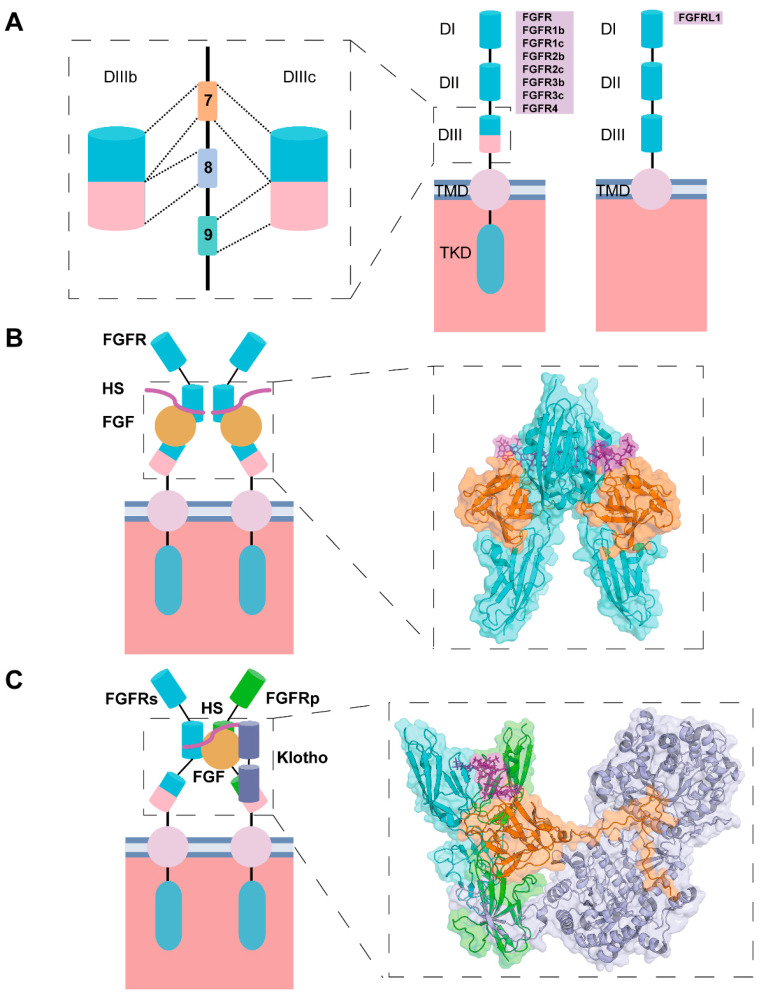
Schematic illustration of FGFRs and FGF-induced FGFR dimerization. (**A**) Structure of FGFRs and FGFRL1, as well as alternative splicing during gene expression. (**B**) Paracrine FGFs (represented by FGF2) promote FGFR dimerization. (**C**) Endocrine FGFs (represented by FGF23) promote FGFR dimerization. Structure diagrams of FGF2-FGFR1c-HS (PDB ID: 1FQ9) and FGF23-FGFR1c-HS-aKlotho (PDB ID: 7YSH). FGFs are colored in orange; FGFR in light blue; Klotho in light purple; and HS in purple. In the FGF23-FGFR1c-HS-aKlotho complex, the primary FGFR (FGFRp) is colored in green, and the secondary FGFR (FGFRs) in light blue.

Paracrine and endocrine FGFs exert a variety of biological functions by activating four major tyrosine kinase receptors (FGFR1, FGFR2, FGFR3, and FGFR4), as well as a truncated receptor lacking the intracellular tyrosine kinase domain (FGFRL1), thereby regulating downstream signal transduction [69]. FGFRs belong to the single-pass transmembrane protein family and are composed of an extracellular domain (ECD), a transmembrane domain (TMD), and an intracellular tyrosine kinase domain (TKD) [70]. The extracellular domain consists of three immunoglobulin-like subunits (DI, DII, and DIII), among which DII and DIII play key roles in ligand binding and receptor dimerization [71]. In FGFR1–3, alternative splicing of exons 7–9, which encode the DIII region, occurs during transcription, resulting in the b isoforms (FGFR1b–3b) encoded by exons 7 and 8, and the c isoforms (FGFR1c–3c) encoded by exons 7 and 9 [72] (Figure 2A)**.** This selective splicing alters the amino acid composition at the ligand-binding pocket, thereby affecting ligand specificity. Furthermore, since FGFR isoforms are differentially distributed across various tissues and cell types, alternative splicing serves as an important mechanism by which FGFs achieve tissue-specific regulation [73].

**Figure 3:**
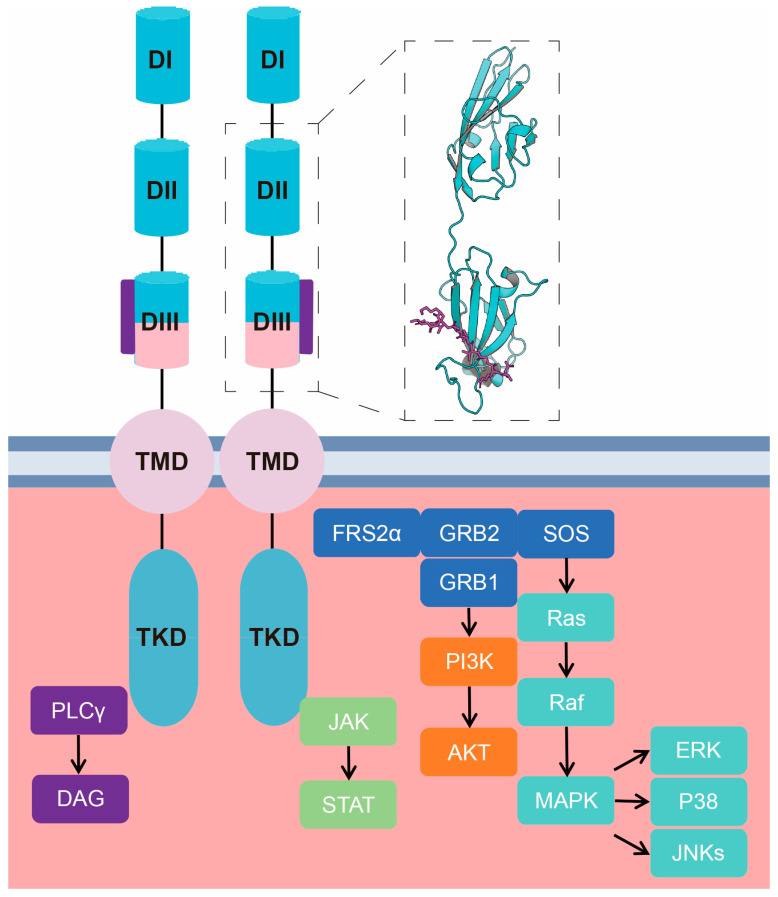
Schematic illustration of the molecular basis and signaling pathways underlying the functional activity of FGF-derived peptides [85,86]. The dashed box shows the binding of FGF-derived peptides to FGFRs.

Activation of the FGFR signaling pathway depends on receptor dimerization [74]. Paracrine FGFs induce receptor dimerization by stably interacting with FGFRs through binding to heparan sulfate [75] (Figure 2B). In contrast, the binding of endocrine FGFs to their receptors requires the synergistic action of heparan sulfate and Klotho family proteins (such as α-Klotho or β-Klotho) to enhance their affinity for FGFRs [76,77] (Figure 2C). FGF-FGFR signaling is transduced through the activation of four classical downstream pathways: (1) the MAPK/ERK pathway, which regulates cell proliferation and differentiation [78]; (2) the PI3K/AKT pathway, which promotes cell survival [79]; (3) the PLCγ/DAG pathway, which regulates cell migration [80]; and (4) the JAK/STAT pathway, which is involved in immune responses, tumor invasion, metastasis, and other processes [81], thereby mediating the biological functions of FGFs [82,83,84] (Figure 3, modified from [69,84]).

The concept of FGF-derived peptides originates from researchers’ efforts to explore the regulation of the FGF-FGFR signaling pathway, aiming to achieve therapeutic potential through targeted modulation of FGF biological functions. Specifically, FGF-derived peptides can act as targeted agonists by directly binding to FGFRs, thereby inducing receptor dimerization and activating downstream signaling pathways. At the same time, they may also function as competitive ligands, inhibiting the binding of natural FGFs to FGFRs and thus blocking the activation of the FGF-FGFR signaling pathway. Based on this concept, the development of FGF-derived peptides is primarily achieved through two strategies: The first strategy utilizes molecular docking software to analyze the key amino acid sequences at the FGF-FGFR interaction interface, allowing for the screening of derived peptides from natural FGFs (Table 1). The second strategy is based on the molecular structures of FGFs and FGFRs, employing high-throughput screening methods such as phage display technology to obtain artificial peptide sequences with high affinity [55,65,87]. However, due to the limitations of current phage libraries [88], such derived peptides typically consist of 6 to 12 amino acid residues. It is noteworthy that shorter peptide sequences may contain multiple FGFR binding sites or even interact with other receptors, thereby conferring novel biological functions. Therefore, the functional characteristics of FGF-derived peptides need to be systematically validated using various in vitro and in vivo experimental models to clarify their specificity and potential off-target effects.

## 3. Activation Functions of FGF-Derived Peptides

FGFs constitute a family of proteins that play critical roles in tissue development, metabolic regulation, and injury repair. Based on the biological functions of FGFs, researchers have designed and developed a series of FGF-derived peptides with similar activities to explore their potential applications in regenerative medicine and drug development.

### 3.1. FGF-Derived Peptides from Natural Sequences

**Osteogenic Differentiation:** The peptides derived from FGF2 have shown significant application prospects in bone and cartilage regeneration. Among them, the FGF2-derived peptide FP2 can activate the AKT and ERK signaling pathways by upregulating the phosphorylation levels of FGFR1 and the adaptor protein FRS2α, thereby significantly enhancing the proliferation and colony-forming ability of human umbilical cord Wharton’s jelly mesenchymal stem cells (hWJ-MSCs). At the same time, injecting hWJ-MSCs pre-treated with FP2 into the joint can effectively alleviate the arthritis symptoms of mice in the osteoarthritis model, demonstrating its good therapeutic potential [29]. Moreover, the FGF2-derived peptides F36 and F77 can also promote the differentiation of human bone marrow mesenchymal stem cells (hBM-MSCs) into osteoblasts [24]. It is worth noting that the short peptides F105 and F119 derived from FGF2 not only have strong heparin-binding ability but also can promote the differentiation of hBM-MSCs into osteoblasts in a dose-dependent manner and accelerate matrix mineralization [39].

**Muscle Regeneration:** Skeletal muscle satellite cells (SCs) are crucial stem cells during the muscle regeneration process and play a key role in the repair and regeneration of muscle fibers [89]. The FGF2-derived peptide-33 and its analogue peptide-33-13 can significantly promote the proliferation of mouse SCs. Pax7 is an important marker gene for maintaining the stemness of SCs, and its expression level directly influences the proliferation and survival of SCs. Studies have shown that peptide-33-13 can also significantly increase the proportion of cells with high expression of Pax7 in proliferating SCs [23]. These results indicate that FGF-derived peptides have significant potential in maintaining the stemness of SCs and promoting muscle regeneration.

**Wound Repair:** FGF-derived peptides also demonstrate broad application prospects in the field of wound repair. The FGF2-derived peptide FP2 can induce hWJ-MSCs to secrete large amounts of exosomes (FP2-exo). These exosomes can not only significantly promote the migration of human dermal fibroblasts but also effectively inhibit the inflammatory response induced by lipopolysaccharide (LPS) [90]. In addition, the FGF7-derived peptide KGFp can promote the differentiation of mouse BM-MSCs into keratinocytes by activating the ERK1/2, STAT3 and AKT signaling pathways. This peptide has high stability in the skin microenvironment. And, when used in combination with stem cells, it can significantly increase the closure rate of wounds in diabetic mouse models (79.3%) while also enhancing the hardness of the skin tissue [46]. These characteristics make KGFp a potential candidate drug for treating chronic wounds, such as diabetic foot ulcers.

**Treatment of Multi-Organ Diseases:** The FGF2-derived peptide FGF-P has shown remarkable efficacy in the prevention and treatment of acute radiation syndrome (ARS). FGF-P can significantly reduce sepsis and bleeding symptoms in animals with radiation-induced bone marrow syndrome and can also effectively alleviate their gastrointestinal inflammation and skin syndromes [32,33,34]. To further enhance the activity of this derivative peptide, researchers linked FGF-P with the heparin-binding sequence (RKRLDRIAR [91]) to develop a new active peptide F2A4-K-NS. This peptide exhibits similar angiogenic-promoting ability to FGF2 in both in vivo and in vitro vascularization models [35]. It not only promotes cell migration, proliferation, and gelatinase secretion but also significantly alleviates ulcerative colitis induced by dextran sulfate sodium (DSS) [36]. Furthermore, other researchers also report on peptide amphiphiles that incorporate FGF-P and peptide domains that drive its self-assembly into supramolecular nanoribbons. These FGF2-PA nanoribbons can significantly promote the proliferation and migration of human umbilical vein endothelial cells (HUVECs) by activating the FGFR1 signaling pathway. And it has the same extent as the native FGF-2 protein at certain concentrations [37].

**Treatment of Neurodegenerative Diseases:** In order to explore the neuroprotective potential of FGF2-derived peptides, researchers used an in vitro oxygen–glucose deprivation (OGD) model and an in vivo rat retinal ischemia–reperfusion (I/R) injury model to evaluate the neuroprotective effect of FGF2-derived peptide FK18 [30]. The results showed that FK18 significantly increased the viability of and attenuated the apoptosis of SH-SY5Y cells. It also markedly alleviated I/R-induced retinal neuronal apoptosis, damage to retinal ganglion cells (RGCs), and morphological and functional damage to the retina. In terms of the molecular mechanism, FK18 could activate the AKT signaling pathway under both physiological and pathological conditions, inhibit the mitochondrial translocation of the proapoptotic protein Bad, increase the expression of Bcl-2/Bax, and reduce the release of cytochrome c in mitochondria. Additionally, there are studies that have reported a series of short peptides, canofins. They are designed based on the interaction structure of FGF2 and FGFR1. They can promote neural synaptic growth by activating FGFRs and exert neuroprotective effects [22]. More importantly, canofins can also inhibit the receptor phosphorylation induced by FGF2, indicating that they have a unique mechanism of action as FGFR agonists.

The F3 region of neural cell adhesion molecule (NCAM) can bind to FGFRs and promote neurite outgrowth [92]. Studies have found that the sixth and seventh β-strands of FGFs are highly homologous to the sequence of this region. Based on this, researchers designed a series of short peptides called hexafins consisting of 16 amino acid residues [20]. Among these, hexafin1/2/3/8/9/10/17 can bind to FGFRs and activate their phosphorylation. Mechanistic studies revealed that basic and hydrophobic amino acids in hexafins play a key role in the binding with FGFRs. Unlike natural FGFs, the binding of hexafins to FGFRs can be inhibited by heparin. Functional experiments further confirmed that hexafin1/2/3/8/9/10/17 can induce neurite growth of cerebellar granule neurons, and the tetramer hexafin 10 has 1250 times higher activity than the monomer. In addition, hexafin1/3/9/10/17 can significantly increase the survival rate of cerebellar granule neurons. These findings suggest that Hexafins are expected to become candidate peptide drugs for regulating neural system functions through the FGFR pathway.

FGF-derived peptides can also be conjugated with other bioactive peptides to exert synergistic pharmacological effects. Researchers integrated the FGF2-derived peptide FGF-P and the laminin signaling peptide IKVAV at the termini of peptide amphiphiles (PAs) with different alkyl tails, constructing a biologically active scaffold. In a severe spinal cord injury mouse model, this scaffold significantly promoted angiogenesis, axonal regeneration, myelin formation, survival of motor neurons, and recovery of neurological function, demonstrating excellent therapeutic potential [38].

**Metabolic Regulation**: FGF23 is a crucial endocrine factor that plays a significant role in regulating phosphate homeostasis in the body [93]. Studies have shown that a short peptide derived from the C-terminal sequence of FGF23 can significantly reduce the phosphate levels in the serum of FGF23 knockout mice, and their efficacy is comparable to that of direct administration of FGF23. This finding indicates that this short peptide has the potential to be a new candidate drug for treating hyperphosphatemia, such as that occurring in chronic kidney disease. It is worth noting that, since this short peptide lacks the binding domain for FGFRs, it may function through non-classical signaling pathways or directly regulate related metabolic processes within cells [50]. Additionally, researchers have successfully constructed a 26-amino acid residue derived peptide PFNP by fusing the nuclear localization signal peptide of FGF1 with a cell membrane-permeable peptide (sequence: AAVALLPAVLLALLAP [94]). In vitro experiments have shown that this short peptide can stimulate DNA synthesis in cells but does not promote their proliferation [95]. Further mechanistic studies have revealed that PFNP does not bind to FGFRs and does not promote their phosphorylation, suggesting that the function of PFNP in stimulating DNA synthesis may be independent of the Ras signaling pathway [19]. Interestingly, PFNP also has the characteristic of significantly inhibiting animal appetite [96]. These research results all indicate that short peptides derived from endocrine FGFs have broad application prospects in the treatment of metabolic-related diseases such as chronic kidney disease and obesity.

### 3.2. Artificially Designed FGF-Derived Peptides

**Wound Repair:** The synthetic short peptide H1, identified through phage display technology, can specifically bind to FGFR2c and exhibit significant biological activity [56]. Studies have demonstrated that H1 promotes angiogenesis and migration of endothelial cells and accelerates the healing of full-thickness skin wounds in rats. Mechanistic investigations revealed that H1 activates the PI3K-AKT and MAPK-ERK1/2 signaling pathways, markedly upregulating the secretion of vascular endothelial growth factor (VEGF), thereby accelerating angiogenesis in the chick embryo chorioallantoic membrane model. Meanwhile, another short peptide, C19jun, which mimics the function of FGF2, is also capable of binding to FGFRs and inducing their dimerization. Further studies revealed that the mechanism of action of this peptide closely resembles that of natural FGF2, and its activation of the FGFR pathway similarly requires the assistance of heparin [52]. The artificially designed peptide FAP1, based on the crystal structure of the FGF2-FGFR1 complex, can promote the proliferation and migration of NIH3T3 cells. In vivo experiments demonstrated that FAP1 binds to FGFR1 to enhance collagen synthesis and promote the migration and proliferation of keratinocytes and fibroblasts, thereby significantly improving wound healing in diabetic mice [51].

**Treatment of Neurodegenerative Diseases:** The artificially designed short peptide CH02 can bind to and activate the FGFR2 signaling pathway while also exhibiting affinity for other FGFR subtypes [55]. In vitro experiments confirmed that CH02 can maintain the survival of sensory neurons and promote axonal growth. Animal studies showed that CH02 significantly promotes nerve regeneration and the recovery of sensory and motor functions in rats with dorsal root injuries. Mechanistic studies demonstrated that CH02 exerts its neuroprotective and regenerative effects by activating the AKT, ERK, and mTOR signaling pathways downstream of FGFRs. These findings provide important evidence for the potential application of CH02 in the treatment of neurodegenerative diseases.

**Metabolic Regulation:** FGF21 has attracted considerable attention due to its significant fat-reducing and glucose-lowering effects [97]. FGFR1c, together with β-Klotho, forms the core complex mediating the physiological functions of FGF21 [98]. Based on the structure of this complex, the agonist peptide F91-8A07 was designed, which can undergo self-dimerization through PEGylation. In vitro experiments demonstrated that this dimer exhibits superior lipid-lowering activity in primary human adipocytes compared to native FGF21. Moreover, F91-8A07 significantly upregulates the expression of Egr-1, a target gene of FGFR1c, in mice. These results suggest that this short peptide has potential application value in the treatment of obesity and diabetes [54].

## 4. Antagonistic Effects of FGF-Derived Peptides

In certain human diseases, abnormally elevated levels of FGFs within tissues may lead to excessive proliferation of target cells and disruption of metabolic homeostasis [99]. To address this, researchers have designed FGF-derived peptides based on the molecular structures and interaction mechanisms of FGFs and their receptors. By inhibiting the binding of ligands to receptors, these peptides offer novel strategies for disease treatment.

### 4.1. FGF-Derived Peptides from Natural Sequences

**Cancer Therapy:** The FGF2-derived peptide FREG specifically binds to the FGF2 molecule and reduces its bioactivity by competitively inhibiting the interaction between FGF2 and FGFRs [25]. Recent studies indicate that FREG can significantly suppress the proliferation, invasion of rat aortic smooth muscle cells and pulmonary metastasis of melanoma cells by the platelet-derived growth factor receptor-α (PDGFR-α) signaling pathway. Acute toxicity experiments confirmed the high safety profile of FREG, as even high doses did not induce pathological damage to liver, kidney, or lung tissues [26].

Angiogenesis inhibition is a crucial strategy in cancer therapy. A short peptide, 8b-13, designed based on the gN helix of the FGF8b molecule, can interfere with the binding of FGF8b to FGFRs, thereby inhibiting its bioactivity [47,48,49]. This peptide shows significant therapeutic potential in treating various cancers characterized by aberrant expression of FGF8b, such as prostate and breast cancer.

A cyclic short peptide, P2, designed based on the FGF1 sequence, can both promote the proliferation of BALB/c 3T3 cells and inhibit the interaction between FGF1 and FGFRs through competitive binding. The structural analysis of Nuclear magnetic resonance (NMR) results indicates that the specific amino acid in P2 and its characteristic cyclic conformation are crucial for maintaining its function [21]. A similar situation is that the linear short peptide BGF2, designed based on FGF2 and its cyclic derivative BGF1, which is formed through disulfide bonds, also exhibits different biological activities. The research shows that BGF1 can significantly inhibit the proliferation of HUVECs, 4T1 breast cancer cells, U87 glioblastoma cells, and SKOV3 ovarian cancer cells induced by FGF2, while the linear structure of BGF2 has no such effect [40]. This indicates that the molecular structure of FGF-derived peptides also has a certain degree of influence on the exertion of their biological functions.

The FGF2-derived peptide P5 and its cyclic derivative DcP5 can all inhibit the activation of the FGFR2 signaling pathway mediated by FGF2 to varying degrees [27]. P5 can inhibit the proliferation of DU145 prostate cancer cells in a FGFR2-dependent manner and significantly delay tumor growth in animal models. In contrast, since the cyclic DcP5 has a stronger binding affinity to FGFR2 and is more stable in the body, it can further enhance its anti-cancer activity. Additionally, some researchers have combined P5 with hyaluronic acid (HA) to prepare nanoparticles HA-P5. It can effectively repair acne lesions by inhibiting the FGFR signaling pathway, and its therapeutic effect is superior to the commercially available FGFR inhibitor AZD4547 [28].

Based on the FGF2 sequence and introducing cysteine modification at its C-terminus, the derived peptide bFGFp also has the ability to inhibit the binding of FGF2 to FGFR1. When researchers coupled bFGFp with bovine serum albumin (BSA) and maleimide-polyethylene glycol-phosphatidylethanolamine (Maleimide-PEG-PE), they obtained bFGFp-BSA and bFGFp-liposomes. These can not only efficiently bind to FGF2 but also be specifically taken up by NIH3T3 cells with high expression of FGFRs and do not induce cell proliferation [41]. After modifying it with methoxy polyethylene glycol-distearylphosphatidylethanolamine (mPEG-DSPE), this effect was further enhanced [100].

Active immunization targeting FGFs is an emerging direction in tumor immunotherapy. Researchers coupled 13 derivative peptides based on FGF2 with recombinant hepatitis B core antigen (HBcAg) virus-like particles (VLPs) and immunized mice. The experimental results showed that this method could induce mice to produce high-titer FGF2-specific antibodies and significantly increase the level of interferon-γ (IFN-γ) in the serum. At the same time, the number of immune effector cells (such as CD8+ IFN-γ+ cells, cytotoxic T lymphocytes, and CD4+ IFN-γ+ Th1 cells) in the mice’s bodies increased significantly after immunization. While the number of immunosuppressive cells (such as CD4+ CD25+ FOXP3+ Treg cells and Gr-1+ CD11b+ myeloid-derived suppressor cells) decreased significantly [31]. This immunization strategy provides an important reference for the development of new tumor immunotherapies.

**Hair Follicle Repair:** The growth process of hair follicles includes the anagen phase, catagen phase and telogen phase. This cyclical change plays an important role in the normal growth of hair [101]. Studies have shown that FGF5 is significantly highly expressed in the later stage of the active growth phase of hair follicles, and it promotes the transformation of hair follicles from the active growth phase to the regressive phase [102]. To inhibit the activity of FGF5, researchers based on the structure of FGF5 developed a derivative peptide P3 consisting of 10 amino acid residues. This peptide can effectively inhibit the proliferation of NIH3T3 and BALB/c 3T3 cells induced by FGF5 and can alleviate the inhibitory effect of FGF5 on the length of hair follicles and the growth of hair cells. These results indicate that P3 has potential application value for anti-hair loss and promoting hair growth [45].

### 4.2. Artificially Designed FGF-Derived Peptides

**Cancer Therapy:** The application of phage display technology in cancer-targeted therapy is becoming increasingly widespread. Through this technology, peptide antagonists that can specifically bind to target proteins can be screened out [103]. For example, the artificially synthesized peptide AP8, designed using this approach and capable of specifically binding to FGF1, can block the activation of the ERK1/2 and AKT signaling pathways in breast cancer cells and vascular endothelial cells induced by FGF1. And, by inhibiting the expression of proliferating cell nuclear antigen (PCNA) and Cyclin D1, it can cause the cell cycle to be arrested at the G_0_/G_1_ phase [61]. Clinical studies have shown that the level of FGF2 in the serum of breast cancer patients is significantly elevated [104]. The derivative peptide P7, obtained by screening using the phage surface display technology and specifically binding to FGF2, can also block the activation of the ERK and P38 signaling pathways, effectively inhibiting the proliferation of breast cancer cells induced by FGF2 [62]. In addition, P7 can also inhibit the promotion effect of FGF2 on the transformation of epithelial ovarian cancer cells from the G_0_/G_1_ phase to the S phase and suppress the expression of Cyclin D1 and the activation of the MAPK and AKT signaling pathways, thereby playing a role in the treatment of ovarian cancer [63]. At the same time, P7 can also act as a chemosensitizer, improving the resistance of colorectal cancer patients to irinotecan hydrochloride (CPT-11) [64]. Moreover, the truncated form of P7, P7^Δ^, which is an artificially synthesized peptide with high homology to the DIII domain of FGFR1c and FGFR2c, also shows high affinity for FGF2 and can inhibit the proliferation of cells induced by FGF2 and the formation of new blood vessels [65].

Abnormally high expression of FGF3 has been observed in a large number of breast cancer patients [105]. The specific derivative peptide FP16, designed against FGF3, can inhibit the expression of Cyclin D1 and PCNA, causing the cell cycle to be arrested at the G_0_/G_1_ phase, thereby inhibiting the proliferation of cancer cells caused by FGF3 overexpression [66]. FGF8b is considered a potential therapeutic target for prostate cancer [106]. The artificially synthesized peptide P12, which is highly homologous to the DIII domain of FGFR3c, can also inhibit the expression of PCNA and Cyclin D1, thereby blocking the activation of ERK1/2 and AKT pathways in prostate cancer cells and vascular endothelial cells [67]. The overexpression of FGF9 is closely related to the occurrence of gastric cancer and bladder cancer [107,108]. The derivative short peptide P4, which is highly homologous to the extracellular domain of FGFR3c, can inhibit the proliferation, migration and invasion of tumor cells induced by FGF9, and can increase the sensitivity of gastric cancer cells to chemotherapy drugs [68]. In addition, the derivative peptide F8 designed for the FGFR1-FGFR1 complex can reduce the proliferation efficiency of BA/F3 cells (highly expressing FGFR1c) induced by FGF1 by more than 40%, showing its potential therapeutic value in FGFR1-overexpressing tumors [53].

**Osteogenic Differentiation:** Mutations in the FGFR3 gene can lead to various human skeletal dysplasia syndromes, including achondroplasia, hypochondroplasia, and thanatophoric dysplasia [109]. The highly specific derivative peptide P3 developed based on the extracellular domain of FGFR3 can effectively inhibit the abnormal activation of the FGFR3 pathway and its downstream tyrosine kinase activity. Moreover, P3 can also alleviate the bone growth disorders caused by the lethal chondrodysplasia type II disease [57].

**Metabolic Regulation:** The derivative peptide 23-b6 designed for the FGFR-Klotho complex can act as an antagonist of FGF23. It can alleviate the metabolic disorders caused by abnormal phosphate uptake of FGF23 by inhibiting the ERK pathway and upregulating the expression of type II sodium phosphate cotransporter proteins (NaPi-2a and NaPi-2c) in the opossum kidney cells [59].

**Hair Follicle Repair:** FGF18 is highly expressed during the hair follicle resting phase and plays an important role in regulating the hair follicle growth cycle [110]. The short peptide GPIGS, obtained through functional screening of bacterial conditioned medium, exhibits the effect of promoting hair regeneration in mice [111]. Subsequent studies revealed that the dipeptide derived from this short peptide has similar receptor specificity to FGF18 and can activate FGFR1c, FGFR3c and FGFR4 while competitively inhibiting the regulatory effect of FGF18. This dipeptide also has great potential in regulating skin physiological functions, promoting hair growth and accelerating wound healing [60].

## 5. Drug Delivery

To enhance the transfection efficiency of the target gene, researchers constructed a novel gene transfection vector by coupling the nuclear localization signal peptide of FGF3 with the polyamidoamine (PAMAM) dendrimers [42]. The experimental results showed that it was significantly superior to similar polyethyleneimine (PEI) materials in terms of cell transfection efficiency and had extremely low cytotoxicity. Additionally, the derivative peptide K16SP, which fused 16 lysine residues (K16) with the FGF4 signal peptide (SP), could efficiently and non-covalently deliver complete macromolecular proteins (such as immunoglobulin G, β-galactosidase, and green fluorescent protein) into mammalian cells [43]. There are also reports that, when the molecular complex composed of FGF4 signal peptide and polylysine is combined with fluorescently labeled oligonucleotides (ONs), this complex can significantly enhance the cellular uptake of ONs in a dose-dependent and non-endocytic manner [44]. Similarly, when the short peptide targeting FGFR is coupled with the polyethyleneimine–polyethylene glycol (PEI-PEG) complexes, this conjugate also exhibits excellent DNA delivery capabilities [58]. These research findings indicate that the short peptides derived from FGFs have significant potential value in the development of efficient and low-toxicity drug delivery systems.

## 6. Conclusions and Future Perspectives

FGFs are a class of key signaling molecules that regulate body development, metabolic homeostasis and tissue repair in the human body. However, natural FGFs are limited in clinical application due to their high production cost, poor stability and complex delivery requirements. In contrast, FGF-derived peptides, as functional alternatives to natural FGFs, have shown broad application potential in the fields of tissue repair, regenerative medicine, disease treatment and new technology development at present.

**Figure 4:**
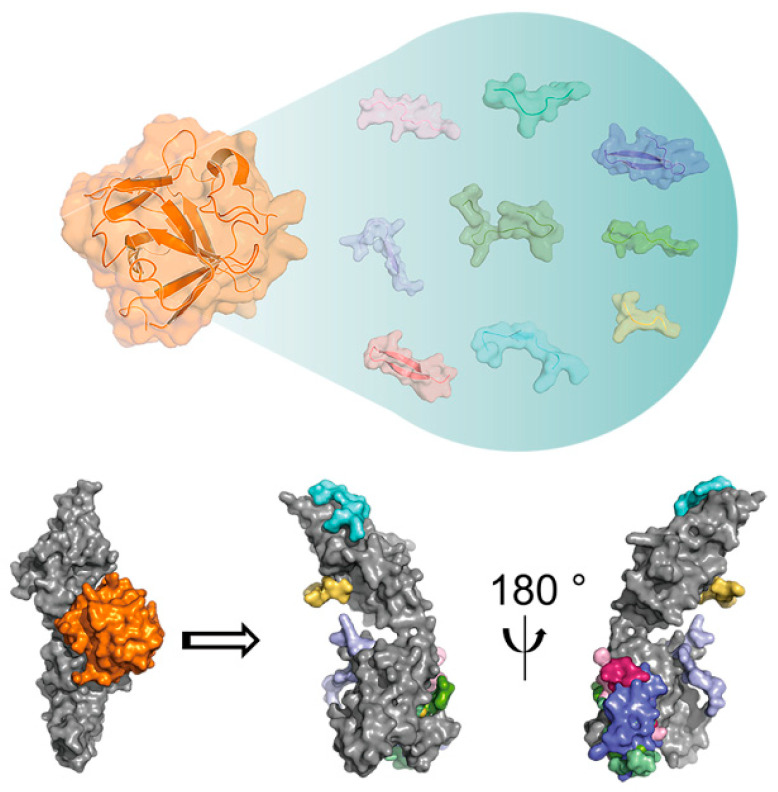
Schematic representation of the binding interaction between FGF-derived peptides and FGFR. Full-length FGF is colored in orange; various FGF-derived peptides are colored in pink, light red, yellow, blue, purple, light purple, green, dark green and light green, respectively; FGFR is colored in grey.

Although these FGF-derived active short peptides currently lack significant sequence homology or structural similarity, they still possess the ability to effectively activate FGFRs and their downstream signaling pathways, ultimately achieving biological functions similar to those of natural FGFs. Interestingly, upon reviewing the literature, we discovered that the functional short peptides derived from specific FGFs (such as FGF2), although containing only 5 to 25 amino acid residues, were able to collectively span almost the entire amino acid sequence of the corresponding full-length protein [20,22,23,24,25,26,27,28,29,30,31,32,33,34,35,36,37,38,39,40,41] (Table 1). This observation suggests that FGFs may exert their biological activities not only in their full-length form but also through combinations of shorter functional peptides. Indeed, pharmacokinetic studies have shown that FGFs generally possess a short in vivo half-life and are readily hydrolyzed by various endogenous proteases into peptide fragments of different lengths [112,113,114]. These findings support the hypothesis that FGF-mediated biological functions can be achieved through multiple molecular forms, encompassing both the intact proteins and their constituent peptide assemblies. In addition, these hydrolyzed peptides can reach lesion sites that are difficult for full-length proteins to access, performing regulatory functions (Figure 4). Therefore, systematically mining active short peptides from FGFs and clarifying their mechanisms of action is a research direction worthy of in-depth exploration.

It is worth noting that the biological activity of FGF-derived peptides is usually achieved through the FGF-FGFR pathway, but some of these peptides’ activities do not rely on the activation of FGFRs [19,23]. This multiple regulatory mechanism enables FGF-derived peptides to retain their disease-treatment capabilities while avoiding the toxic side effects caused by the activation of FGFRs [115]. In addition, due to their small molecular weight and low immunogenicity, FGF-derived peptides also show promising application prospects in the treatment of neurological diseases.

The biological activity assessment of FGF-derived peptides is a key part of their preclinical research. Currently, the detection methods mainly rely on various cell lines and animal models (Table 1). However, due to the diversity of the reported model types and drug dosages at this stage, a unified standard has not yet been established. Researchers need to select appropriate models based on specific research goals and mechanisms of action. Compared with full-length FGFs, FGF-derived peptides can partially or fully simulate the biological functions of FGFs by regulating the FGF-FGFR signaling pathway and may also activate other signaling pathways [19,23], showing new biological activities. Therefore, the selection of activity detection methods should comprehensively consider the target of the derived peptide, expected function and research background. It should be noted that the effective working concentration of FGF-derived peptides in in vitro and in vivo experiments is usually 2 to 3 orders of magnitude higher than that of full-length FGFs. This may be related to their smaller molecular weight, simplified structure and changes in the binding sites with FGFRs [46]. Until now, the structural basis underlying the biological activity of these peptides remains unclear. Therefore, it is necessary to investigate whether these peptides may have off-target effects or cause side effects different from those of the natural FGF protein. Meanwhile, optimizing activity in conjunction with standardized risk assessment will support both the development of FGF-derived peptide medicines and their clinical translation.

Although FGF-derived peptides exhibit good stability and ease of storage in vitro, their in vivo application still faces challenges such as rapid degradation and short half-life. Studies have shown that chemical modifications can significantly improve their stability. For example, cyclization of short peptides can enhance molecular rigidity and increase resistance to proteolytic degradation [116]; N-terminal methylation can improve resistance to biodegradation [117]; and C-terminal amidation (introduction of an -NH_2_ group) can further enhance proteolytic stability [118]. In addition, polyethylene glycol (PEG) modification not only inhibits proteolytic hydrolysis but also improves the biodistribution and solubility of peptides, reduces renal clearance, and prolongs the in vivo half-life [119]. These modification strategies provide technical support for the clinical development of FGF-derived peptides.

The efficient large-scale production of FGF-derived peptides is a prerequisite for their clinical translation. Currently, solid-phase peptide synthesis (SPPS) is the mainstream method for producing short peptides [120]. This technique uses individual amino acids as raw materials and prepares pure peptides through coupling, cleavage, and purification steps, ultimately obtaining stable products through freeze-drying [121]. Therefore, it is suitable for the large-scale production of short FGF-derived peptides. For long FGF-derived peptides, gene engineering methods are usually adopted, with recombinant expression in *E. coli* or yeast expression systems for preparation [122,123]. In recent years, semi-synthetic strategies combining chemical and biological synthesis have gradually emerged [124,125,126]. This approach can obtain polypeptide precursors with specific tags through microbial expression systems and then prepare target peptides through in vitro enzymatic or chemical modification. This scheme can significantly improve the large-scale production efficiency of derived peptides, thereby accelerating their application process in clinical translation.

Recent studies have demonstrated that the therapeutic efficacy of FGF-derived peptides can be further enhanced through their combination with biomaterials [31,37,38,41]. Therefore, in future development, by referring to the established strategies of other bioactive peptides, efforts should be made to integrate FGF-derived peptides with biological materials. A broad range of biomaterials can serve as potential candidates, including lipid-based nanocarriers, hydrogels, chitosan, PEG, dendrimers, and trehalose-based glycopolymers. These can offer multiple advantages: (1) provide protection for peptides against enzymatic degradation and accidental thiol/disulfide bond exchange reactions; (2) extend the peptide’s half-life and reduce adverse reactions; (3) enhance the physical stability of the peptides, including solubility and anti-aggregation properties, and resist environmental pressures; (4) increase topical bioavailability and therapeutic efficacy [127,128,129,130,131]. However, several issues still need to be further addressed clearly, such as the release kinetics of peptides in materials, biocompatibility and stability in various tissues [132,133,134,135].

The development of artificial intelligence (AI) technologies has opened up new directions for the design of new proteins and short peptides [136,137] and has made it possible to design FGF-derived peptides tailored for various clinical applications. Using AI software such as RFDiffusion (v1.1.0), it is now feasible to move beyond traditional strategies based on natural FGF sequence truncation or phage display and to rationally design high-affinity short peptides targeting specific molecules [138]. Subsequent high-throughput functional screening enables the rapid identification of bioactive peptides with desired functions. For example, TGF-β receptor type II (TGF-βRII)-binding peptides designed using tools such as AlphaFold (v2.3.2) and Rosetta (v1.0.1) have demonstrated sub-nanomolar affinity for the receptor and have been applied in cancer therapy [139]. In the future, research into peptide-based therapeutics, including those based on FGFs and other growth factors, is likely to enter an exciting new stage of innovation and application.

In summary, FGF-derived peptides, as novel and efficient therapeutic molecules designed based on native FGFs, demonstrate broad prospects in regenerative medicine, metabolic regulation, and neuroprotection. They overcome the limitations of traditional protein-based drugs and offer new possibilities for precise and effective disease intervention. In the future, with continued advances in chemical modification techniques, scalable production processes, and AI-assisted design, FGF-derived peptides are expected to accelerate the transition from basic research to clinical application, providing innovative solutions for human health.

## Figures and Tables

**Table 1:** Fibroblast growth factor-derived peptides.

Peptide Name [Refs.]	Peptide Sequence	Origin	Corresponding Residues	Working Concentration	Mode of Action	Model	Application
FGF1 NLS [19]	NYKKPKL	Human FGF1	21~27	20~100 μg/mL	fusion with a cell-penetrating peptide	In Vitro Model: NIH3T3 Cell Line	Metabolic Regulation
Hexafin 1 [20]	TGQYLAMDTDGLLYGS	Human FGF1	76~91	0~100 μmol/L	Tetramer	In Vitro Model: Neurite Outgrowth Assay Using Cerebellar Granule Neurons	Neural Functional Recovery
peptide 1 [21]	SKKHAEKNWF	human FGF1	114~123	10~200 μmol/L	Cyclic peptide	In Vitro Model: 3T3 Cell Proliferation Assay	Rational Design of Peptides
Canofin 1 [22]	HFKDPKRLYCK	human FGF2	25~35	0~100 μmol/L	Tetramer	In Vitro Model: Neurite Outgrowth Assay of Cerebellar Granule Neurons	Neural Functional Recovery
Peptide 33 [23]	CKNGGFF	human FGF2	34~40	10~400 μmol/L	Linear monomer	In Vitro Model: Skeletal Muscle Satellite Cell Proliferation Assay	Muscle Regeneration
Peptide 33-13 [23]	CKNGGFFLRIHPD	human FGF2	34~46	10~400 μmol/L	Linear monomer	In Vitro Model: Skeletal Muscle Satellite Cell Proliferation Assay	Muscle Regeneration
F36 [24]	PDGRVD	human FGF2	45~50	100 μmol/L (conjugation dose)	immobilized on the surface of a chitosan membrane	In Vitro Model: Human Mesenchymal Stem Cell Adhesion and Osteogenic Differentiation Assay	Bone Regeneration
FREG [25,26]	DPHIKLQLQAE	human FGF2	57~67	0~100 ng/mL(cells) 3~6 mg·kg^−1^·d^−1^ (animals)	Linear monomer	In Vitro Model: Human Melanoma Cell Proliferation and Invasion Assay In Vivo Model: Melanoma Mouse Model	Tumor Suppression
P5 and DcP5 [27,28]	LQLQAEER	human FGF2	62~69	5~15 μmol/L (cells) 10 mg/kg (animals) 20~200 μmol/L (conjugation dose)	Linear monomer Cyclic peptide conjugated with the polysaccharide hyaluronic acid	In Vitro Model: DU145 Prostate Cancer Cell Proliferation Model In Vivo Models: Tumor Model and Acne Model	Tumor Suppression Alleviation of Acne
FP2 [29]	ERGVVSIKGV	human FGF2	68~77	0.05 μg/mL (conjugation dose)	fused with mussel adhesive protein immobilized on the surface of the culture plate	In Vitro Model: Proliferation and Differentiation of Human Wharton’s Jelly-Derived Mesenchymal Stem Cells In Vivo Model: Osteoarthritis Model	Bone Regeneration
Hexafin 2 [20]	ANRYLAMKEDGRLLAS	human FGF2	79~94	0~100 μmol/L	Tetramer	In Vitro Model: Neurite Outgrowth Assay of Cerebellar Granule Neurons	Neural Functional Recovery
F77 [24]	KEDGRLL	human FGF2	86~92	100 μmol/L (conjugation dose)	immobilized on the surface of a chitosan membrane	In Vitro Model: Adhesion and Osteogenic Differentiation of Human Mesenchymal Stem Cells	Bone Regeneration
FK18 [30]	FFFERLESNNYNTYRSRK	human FGF2	102~119	0~100 μg/mL	Linear monomer	In Vitro Model: Oxygen-Glucose Deprivation (OGD) Model in SH-SY5Y Cells In Vivo Model: Retinal Ischemia Model	Neural Functional Recovery
Peptide 12 [31]	FFFERLESNNYNTYRSRKYSSWYVA	human FGF2	102~126	50 μg/animal	conjugated with VLPs	In Vivo Model: Breast Tumor Model	Tumor Suppression
FGF-P [32,33,34,35,36,37,38]	YRSRKYSSWYVALKR	human FGF2	115~129	200 ng/mL (cells) 0~20 mg/kg (animals) 3~6 mmol/L (conjugation dose) 40~400 ng/sample (fusion protein)	Linear monomer conjugated with PA fused with a heparin-binding sequence	In Vitro Model: Proliferation and Migration Assays Using Hs-27 Fibroblasts and Keratinocytes In Vivo Model: Total Body Irradiation (TBI) Model, Bone Marrow Syndrome Model, Skin Burn Injury Model, Spinal Cord Injury (SCI) Model	Multi-Organ Repair Spinal Cord Injury Repair Tissue Regeneration
F105 [39]	YKRSRYT	human FGF2	120~114	100 μmol/L (conjugation dose)	immobilized on the surface of the culture plate	In Vitro Model: Adhesion and Osteogenic Differentiation of Human Mesenchymal Stem Cells	Bone Regeneration
BGF1 [40]	CLKRTGQYKLC	human FGF2	127~135	0~1.8 mmol/L (cells) 2~10 mg/kg (animals)	Cyclic peptide	In Vitro Model: Proliferation models of human umbilical vein endothelial cells (HUVECs), 4T1 breast cancer cells, U87 glioblastoma cells, and SKOV3 ovarian cancer cells In Vivo Model: 4T1 Breast Cancer Model	Tumor Suppression
bFGFp [41]	KRTGQYKLC	human FGF2	128~135	100 mg/mL (conjugation dose)	conjugated with bovine serum albumin or liposomes	In Vitro Model: 3T3 Cell Proliferation Assay	Tumor Suppression
F119 [39]	KRTGQYKLGSKTGPGQK	human FGF2	128~144	100 μmol/L (conjugation dose)	immobilized on the surface of the culture plate	In Vitro Model: Adhesion and Osteogenic Differentiation of Human Mesenchymal Stem Cells	Bone Regeneration
Canofin 3 [22]	KTGPGQKAIL	human FGF2	138~147	0~100 μmol/L	Tetramer	In Vitro Model: Neurite Outgrowth Assay of Cerebellar Granule Neurons	Neural Functional Recovery
Canofin 2 [22]	FLPMSAKS	human FGF2	147~155	0~100 μmol/L	Tetramer	In Vitro Model: Neurite Outgrowth Assay of Cerebellar Granule Neurons	Neural Functional Recovery
FGF3 NLS [42]	RRRK	human FGF3	44~47	0~100 μg/mL	conjugated with PAMAM	In Vitro Model: Transfection of HEK293 and HeLa Cells	Cell Transfection
Hexafin 3 [20]	SGRYLAMNKRGRLYKS	human FGF3	93~108	0~10 μmol/L	Tetramer	In Vitro Model: Neurite Outgrowth Assay of Cerebellar Granule Neurons	Neural Functional Recovery
SP and IP [43,44]	AAVALLPAVLLALLAP	human FGF4	7~22	0~80 μmol/L (conjugation dose) 0~500 μmol/L (conjugation dose)	conjugated with lysine conjugated with PL	In Vitro Model: Protein Delivery into 143B, TE85, MG63, and FOB Cells; Oligonucleotide Delivery into A549 Cells	Protein Delivery Oligonucleotide Delivery
P3 [45]	VGIGFHLQIY	human FGF5	95~104	1~1000 mmol/L (cells) 5 μg/subject (animals)	Linear monomer	In Vitro Model: 3T3 Cell Proliferation Assay In Vivo Model: Depilated Mouse Model	Hair Follicle Repair
KGFp [46]	KELILENHYNTYA	human FGF7	140~152	1~100 ng/mL (cells)	Linear monomer conjugated to a 3D porous scaffold	In Vitro Model: Migration and Differentiation of Human Bone Marrow Mesenchymal Stem Cells In Vivo Model: Chronic Wound Model in Type 2 Diabetic Mice	Tissue Repair
8b-13 [47,48,49]	PNFTQHVREQSLV	human FGF8	30~42	1~125 nmol/L	Linear monomer	In Vitro Model: Proliferation Assay of PC-3 and DU-145 Prostate Cancer Cells	Tumor Suppression
Hexafin 8 [20]	TGLYICMNKKGKLIAK	human FGF8	104~119	0~10 μmol/L	Tetramer	In Vitro Model: Neurite Outgrowth Assay of Cerebellar Granule Neurons	Neural Functional Recovery
Hexafin 9 [20]	SGLYLGMNEKGELYGS	human FGF9	112~127	0~100 μmol/L	Tetramer	In Vitro Model: Neurite Outgrowth Assay of Cerebellar Granule Neurons	Neural Functional Recovery
Hexafin 10 [20]	SNYYLAMNKKGKLYGS	human FGF10	128~143	0~10 μmol/L	Tetramer	In Vitro Model: Neurite Outgrowth Assay of Cerebellar Granule Neurons	Neural Functional Recovery
Hexafin 17 [20]	SEKYICMNKRGKLIGK	human FGF17	93~108	0~10 μmol/L	Tetramer	In Vitro Model: Neurite Outgrowth Assay of Cerebellar Granule Neurons	Neural Functional Recovery
FGF23 Peptide [50]	AEDDSERDPLNVLKPRARMTPAPAS	human FGF23	181~205	0.2 nmol/L	Linear monomer	In Vivo Model: Hyperphosphatemia in *Fgf23^−/−^* Mice	Metabolic Regulation
FAP1 [51]	RERNEVNHYRTY	Computational Design of Peptide Derivatives Targeting Human FGFR1c		1~100 ng/mL (cells) 1~10 mg/kg (animals)	Linear monomer	In Vitro Model: Proliferation and Migration Assays Using NIH 3T3 Cells In Vivo Model: Diabetic Mouse Wound Healing Model	Tissue Repair
C19jun [52]	AESGDDYCVLVFTDSAWTKICDWSHFRN	Phage Display Technology for Screening FGFR1c-Binding Peptide Derivatives		0~10 nmol/L	fused with human c-Jun residues for expression Dimer	In Vitro Model: Swiss 3T3 Cell Proliferation and Neurite Outgrowth in Neuronal Cells	Tissue Repair
F8 [53]	ACSLNHTVNC	Phage Display Technology for Screening FGFR1c-Binding Peptide Derivatives		0~10 μmol/L	Cyclic peptide	In Vitro Model: BA/F3 Cell Proliferation Assay	Tumor Suppression
F91-8A07 [54]	LPGRTCREYPDLWWVRCY	Phage Display Technology for Screening FGFR1c/β-Klotho-Binding Peptide Derivatives		0~1000 μmol/L (cells) 0~1000 nmol/kg (animals)	Dimer	In Vitro Model: Primary Human Adipocyte Model In Vivo Model: Mouse Model	Metabolic Regulation
CH02 [55]	GPANVET	Phage Display Technology for Screening FGFR2c-Binding Peptide Derivatives		0~40 μmol/L	Linear monomer	In Vitro Model: Neurite Outgrowth Assay of Dorsal Root Ganglion (DRG) Neurons In Vivo Model: Rat Dorsal Root Compression Injury Model	Neural Functional Recovery
H1 [56]	SNFLHLG	Phage Display Technology for Screening FGFR2c-Binding Peptide Derivatives		0~20 μmol/L (cells) 0~1000 μmol/L (animals)	Linear monomer	In Vitro Model: 3T3 Cell Proliferation and Migration Assay In Vivo Model: Full-Thickness Excisional Wound Model	Tissue Repair
P3 [57]	VSPPLTLGQLLS	Phage Display Technology for Screening FGFR3-Binding Peptide Derivatives		0~50 μmol/L (cells) 100 μg·kg^−1^·d^−1^ (animals)	Linear monomer	In Vitro Model: ATDC5 Cell Proliferation and Chondrogenic Differentiation Model In Vivo Model: TDII Mouse Lethal Phenotype Model	Bone Regeneration
peptide [58]	MQLPLAT	Phage Display Technology for Screening FGFR-Binding Peptide Derivatives		10–20 μg/mL (conjugation dose)	Conjugation with PEI-PEG	In Vitro Model: B16F10 Cell Transfection Model	Cell Transfection
23-b6 [59]	SSPPKSP	Phage Display Technology for Screening FGFR-Klotho-Binding Peptide Derivatives		0~0.1 μmol/L	Linear monomer	In Vitro Model: Phosphate Uptake Assay in Renal Proximal Tubule Cells	Metabolic Regulation
Pro-Ile [60]	PI	Functional Screening of Human FGFR-Binding Peptide Derivatives Using Bacterial Conditioned Medium		0~1 mmol/L	Linear monomer	In Vitro Model: Keratinocyte Proliferation Assay	Hair Follicle Repair
AP8 [61]	AGNWTPI	Phage Display Technology for Screening FGF1-Binding Peptide Derivatives		0~16 μmol/L	Linear monomer	In Vitro Model: Proliferation Assay of Breast Cancer Cells and Human Umbilical Vein Endothelial Cells	Tumor Suppression
P7 [62,63,64]	PLLQATLGGGS	Phage Display Technology for Screening FGF2-Binding Peptide Derivatives		0~16 μmol/L (cells)	Linear monomer	In Vitro Model: Proliferation and Migration Assay of MDA-MB-231 Breast Cancer Cells	Tumor Suppression
P7^Δ^ [65]	PLLQATL	Phage Display Technology for Screening FGF2-Binding Peptide Derivatives		0~16 μmol/L (cells) 1 μmol/L (animals)	Linear monomer	In Vitro Model: Proliferation Assay of BALB/c 3T3 Cells In Vivo Model: Chick Embryo Chorioallantoic Membrane (CAM) Assay	Tumor Suppression
FP16 [66]	VLWLKNR	Phage Display Technology for Screening FGF3-Binding Peptide Derivatives		0~16 μmol/L	Linear monomer	In Vitro Model: Proliferation Assay of MDA-MB-231 and T47D Breast Cancer Cells	Tumor Suppression
P12 [67]	HSQAAVP	Phage Display Technology for Screening FGF8b-Binding Peptide Derivatives		0~16 μmol/L	Linear monomer	In Vitro Model: Proliferation Assay of PC-3 and HUVECs	Tumor Suppression
P4 [68]	NVFTVSP	Phage Display Technology for Screening FGF9-Binding Peptide Derivatives		0~16 μmol/L	Linear monomer	In Vitro Model: Proliferation Assay of SGC-7901 Gastric Cancer Cells and RT-112 Bladder Cancer Cells	Tumor Suppression

## Data Availability

No new data were created or analyzed in this study.

## Abbreviations

The following abbreviations are used in this manuscript:

AI	artificial intelligence
ARS	acute radiation syndrome
BSA	bovine serum albumin
CAM	chorioallantoic membrane
DSPE	distearoyl phosphatidylethanolamine
DSS	dextran sulfate sodium
ECD	extracellular domain
FGF	fibroblast growth factor
FGFR	fibroblast growth factor receptor
HBcAg	hepatitis B core antigen
hBM-MSCs	human bone marrow mesenchymal stem cells
HS	heparan sulfate
HUVECs	human umbilical vein endothelial cells
hWJ-MSCs	human Wharton’s jelly mesenchymal stem cells
I/R	ischemia–reperfusion
IFN-γ	interferon-γ
LPS	lipopolysaccharide
NCAM	neural cell adhesion molecule
NLS	nuclear localization signal
OGD	oxygen-glucose deprivation
ONs	oligonucleotides
PAMAM	polyamidoamine
PAs	peptide amphiphiles
PCNA	proliferating cell nuclear antigen
PDGFR-α	platelet-derived growth factor receptor-α
PE	phosphatidylethanolamine
PEG	polyethylene glycol
PEI	polyethylenimine
SCs	skeletal muscle satellite cells
SP	signal peptide
SPPS	solid-phase peptide synthesis
TGF-βRII	transforming growth factor-β receptor type II
TKD	tyrosine kinase domain
TMD	transmembrane domain
VEGF	vascular endothelial growth factor
VLPs	virus-like particles
